# Development of a prognostic nomogram for ocular melanoma: a SEER population-based study (2000–2021)

**DOI:** 10.3389/fmed.2025.1494925

**Published:** 2025-01-29

**Authors:** Miyun Zheng, Maodong Xu, Mengxing You, Zhiqing Huang

**Affiliations:** ^1^Department of Ophthalmology, The First Hospital of Putian City, Putian, China; ^2^The School of Clinical Medicine, Fujian Medical University, Fuzhou, China; ^3^Department of Medical Oncology, The First Hospital of Putian City, Putian, China

**Keywords:** ocular melanoma, machine learning, SHAP, SEER, prognosis, nomogram

## Abstract

**Introduction:**

Ocular melanoma (OM) is a rare but lethal subtype of melanoma. This study develops a prognostic nomogram for OM using machine learning and internal validation techniques, aiming to improve prognosis prediction and clinical decision-making.

**Methods:**

Independent prognostic variables were identified using univariate and multivariate COX proportional hazard regression models. Significant variables were then incorporated into the nomogram. The predictive accuracy of the nomogram was evaluated using receiver operating characteristic (ROC) curves, calibration plots, decision curve analysis (DCA), and 10-fold cross-validation. The performance of the nomogram was compared with that of a machine learning model.

**Results:**

Thirteen variables, including age, sex, tumor site, histologic subtype, stage, basal diameter size, tumor thickness, liver metastasis, first malignant primary indicator, marital status, and treatment modalities (surgery/radiotherapy/chemotherapy) were identified as independent prognostic factors for overall survival (OS) and were included in the nomogram (all *P* < 0.05). The nomogram showed a concordance index of 0.712. The areas under the curve (AUC) for predicting 3-, 5-, and 10-year survival rates were 0.749, 0.734, and 0.730, respectively. Calibration plots for 3-, 5-, and 10-year survival were in close agreement with the ideal predictions, and DCA indicated a superior net benefit. The average AUC from 10-fold cross-validation was 0.725. The machine-learning model identified liver metastasis as the most significant predictor of survival, followed by age, radiotherapy, stage, and other factors that were incorporated into the nomogram. The machine-learning model achieved a predictive AUC score of 0.750.

**Conclusions:**

A robust nomogram incorporating 13 significant clinicopathological variables was developed. The combined use of ROC curve analysis, calibration plots, DCA, 10-fold cross-validation, and machine learning confirmed the strong predictive performance of the nomogram for survival outcomes in patients with OM.

## 1 Introduction

Ocular melanoma (OM) is a rare subtype of melanoma; the incidence rate is about 6.2 per million, accounting for ~5% of all melanoma cases (1). Uveal melanoma (UM), which includes melanomas of the choroid, ciliary body, and iris, is the predominant anatomical site of OM, accounting for ~85% of these cases (2). Despite its rare occurrence, OM is highly lethal owing to its aggressive nature and high potential for metastasis (3). Although primary melanoma can be treated effectively, ~30% of patients experience recurrence that involves metastatic disease. The prognosis of OM is related to many factors, such as clinical, pathological, and genomic characteristics (4–6). However, a reliable prognostic model for predicting the outcome of OM is currently lacking.

Given the high prevalence of UM among OM, delineating independent prognostic indicators and formulating prognostic frameworks for UM is necessary (7–9). Nonetheless, the absence of an optimal prognostic prediction model encompassing all OM subtypes underscores the pressing need for a robust predictive model to elucidate OM prognosis and inform clinical decision-making. Nomograms, which are graphical instruments that assign distinct points to each variable within a predictive model, facilitate the translation of cumulative points into corresponding survival probabilities.

Machine-learning algorithms have emerged as innovative tools that are extensively employed in cancer research. Among them, eXtreme Gradient Boosting (XGBoost) as a prediction gradient boosting tree stands out as a notable ensemble learning technique (10). This method incrementally enhances prediction efficacy by iteratively training a sequence of decision trees. Owing to their ability to manage structured data and capture non-linear associations, gradient-boosting trees outperform random forests in terms of prediction accuracy. SHapley Additive exPlanations (SHAP) constitute a method devised to elucidate the prediction outcomes derived from machine learning models (11). By integrating these two methodologies, we can assess the predictive effectiveness of the model in conjunction with a profound understanding of how each feature shapes the prediction outcome.

In this study, we developed a prognostic prediction nomogram tailored specifically for patients with OM. Given the rarity of OM, external validation of the model using a substantial dataset remains challenging. To comprehensively assess the predictive probability of our model, we used traditional evaluation methods, including receiver operating characteristic (ROC) curves, calibration curves, and decision curve analysis (DCA), along with internal validation techniques. Additionally, we leveraged advanced methodologies, namely, XGBoost, in conjunction with SHAP to conduct a multifaceted evaluation, compare models, and discern the efficacy of the established prognostic model across various dimensions.

## 2 Materials and methods

### 2.1 Study patients

Cohort information for all patients was extracted from the National Cancer Institute Surveillance, Epidemiology, and End Results (SEER) database between 2000 and 2021. SEER^*^Stat software version 8.4.3 was used for data selection which covers ~26.5% of the U.S. population from 17 registries. OMs were defined by the International Classification of Diseases for Oncology, Third Edition (ICD-O-3) site code, C69.0-69.6 or C69.8-69.9, and ICD-O-3 histology codes 8720-8790.

**Figure 1:**
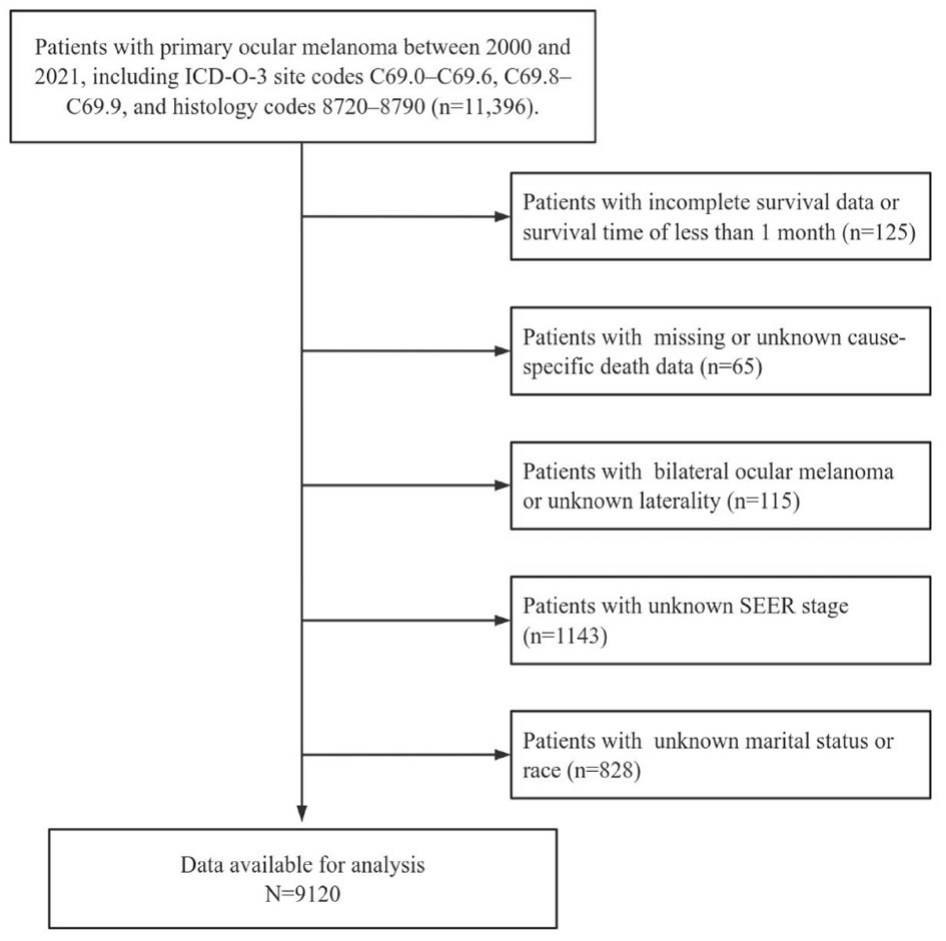
Flow chart of cases selection from SEER database.

The inclusion criteria were as follows: (1) patients diagnosed with primary OM between 2000 and 2021; (2) ICD-O-3 site code of C69.0 (conjunctiva), C69.1 (cornea, not otherwise specified [NOS]), C69.2 (retina), C69.3 (choroid), C69.4 (ciliary body and iris), C69. 5(lacrimal gland), C69.6 (orbit, NOS), C69.8 (overlapping lesion of eye and adnexa), and C69.9 (eye, NOS); (3) ICD-O-3 histology code 8770 (mixed epithelioid and spindle cell melanomas), 8771 (epithelioid cell melanomas), 8772-8774 (spindle-cell melanomas), 8720 (malignant melanoma, NOS) and other (including 8721-8723 [nodular melanoma, balloon cell melanoma and malignant melanoma with regression], 8730 [amelanotic melanoma], 8740-8746 [malignant melanoma in junctional nevus, malignant melanoma in precancerous melanosis, lentigo malignant melanoma, superficial spreading melanoma, acral lentiginous melanoma, desmoplastic melanoma, and mucosal lentiginous melanoma], and 8761 [malignant melanoma in giant pigmented nevus]); and (4) patients with localized, regional and distant tumors defined using SEER staging system (SEER historic stage A and combined summary stage). Patients with unknown survival data or survival duration < 1 month, unknown laterality, SEER stage, race and marital status were excluded. A flowchart of patient selection is shown in Figure 1. Because the study used a de-identified and publicly available database, ethical approval was not required. The design and analysis procedures were performed in accordance with the principles outlined in the Declaration of Helsinki. Additional information regarding the SEER program can be found on the SEER website (https://seer.cancer.gov).

### 2.2 Study variables

The variables used in the SEER database included age, sex, race, site, laterality, histological subtype, combined summary stage, SEER historic stage A, surgery, radiation, chemotherapy, measured basal diameter, measured thickness, metastasis to the liver, metastasis to the lung, first malignant primary indicator (FMPI), marital status, survival months, and vital status. The overall survival (OS) was designated as the outcome-predicting variable, which was measured as the time from diagnosis to death due to any cause.

### 2.3 Establish and validate the nomogram model

To build a nomogram model for predicting prognostic risk factors for ocular melanoma, we initially used a COX proportional hazards regression model. The independent variables were analyzed using univariate COX proportional hazards regression, and those with *P* < 0.05 were included in multivariate COX proportional hazards regression. Subsequently, statistically significant indicators were included in the nomogram model generation. Additionally, hazard ratios (HR) and matched 95% confidence intervals (CI) were recorded.

By incorporating the selected indicator factors, we developed a nomogram to predict the 3-, 5-, and 10-year survival for patients with OM. The concordance index (C-index) served as a metric to gauge the discriminatory capacity of the model, ranging from 0.5 to 1.0. A value of 0.5 signifies random performance, whereas 1.0 denotes perfect alignment between the model predictions and actual outcomes. Additionally, ROC curves and their associated area under the curve (AUC) were used to elucidate the balance between true-positive and false-positive rates across various thresholds. Moreover, calibration curves were used to assess the agreement between the predicted and observed probabilities at the 3-, 5-, and 10-year intervals. DCA was conducted to evaluate the clinical relevance of the model by examining its net benefit across different decision thresholds.

The 10-fold cross-validation involved dividing the dataset into 10 equally-sized subsets. In each iteration, one subset was used for testing, and the remaining nine subsets were used for training. This process was repeated 10 times to ensure that each subset was tested at least once.

### 2.4 Statistical analysis

The X-tile software (Yale University, USA, Version 3.6.1) was used to determine the optimal cutoff value for continuous variables by artificially dividing them into distinct groups. The highest chi-square values calculated by Kaplan–Meier survival analysis and the logarithmic rank test were used as the optimal cutoff values. Based on the results, we divided the age into ≤ 61 years and >61 years. The tumor basal diameter was divided into ≤ 14.9 mm and >14.9 mm, and the measured thickness was divided into ≤ 5.1 mm and >5.1 mm. Patient characteristics, including numbers and proportions, were analyzed using descriptive statistical methods. The Kaplan–Meier method was used to analyze the OS and corresponding survival curves.

XGBoost is a powerful gradient boosting tree algorithm that can be combined with SHAP to obtain more accurate and interpretable prediction models. To analyze the data produced by the machine learning models, we used an explanation model called SHAP to address the problem of black-box predictions. By calculating the SHAP value, the SHAP explanation model measured the impact of each input feature on the predicted output. The values were then used to prioritize the features and visualize important associations. The importance and predictive power of the prognostic predictor increased with higher values. The SHAP analysis utilized the SHAPforxgboost library (V.0.1.3). Finally, the results of the machine learning algorithm were compared with the indicators in the nomogram.

All statistical analyses were performed using RStudio software (V.4.3.1; R Core Team, Vienna, Austria). All statistical tests were two-tailed, and *P* < 0.05 indicated statistical significance.

## 3 Results

### 3.1 Patient characteristics

**Figure 2:**
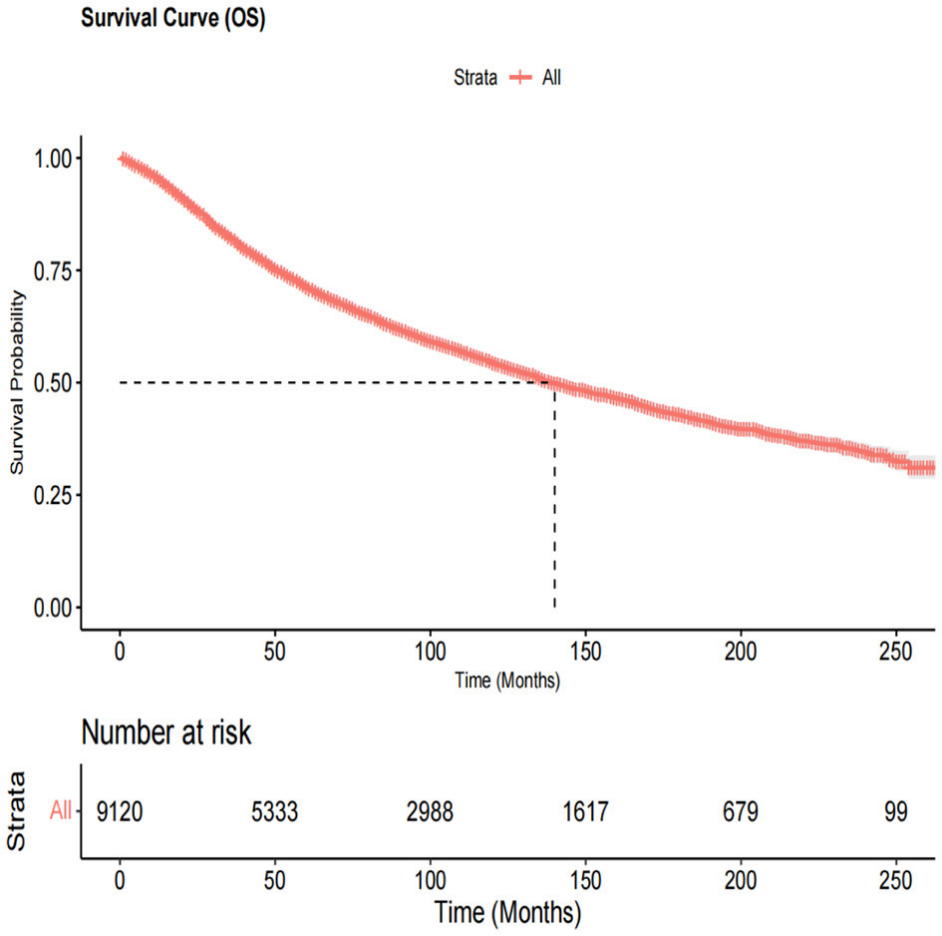
The overall survival curve in all ocular melanoma patients. The median survival time was 132 months.

This study included 9,120 patients [4,329 women (47.5%) and 4,791 men (52.5%)] with primary OM. The demographic and clinicopathological features and treatment information of patients with OM are summarized in Table 1. Among them, the age at OM diagnosis was >61 years in 4,980 patients (54.6%), and the majority of patients were Caucasians (97.4%). The choroid was the most common primary OM site (78.3%), followed by the ciliary body (10.8%). All patients had unilateral lesions with similar rates on both the left and right sides. Based on the histologic subtypes, 7,150 (78.4%) patients were included in the NOS category, 146 (1.6%) were classified as having other types combined as NOS/Other group (80%), 836 (9.2%) had spindle cell melanoma, 715 (7.8%) had mixed epithelioid and spindle cell melanoma, and 273 (3%) had epithelioid cell melanoma. We combined the Combined Summary Stage (2004+) and SEER historical stage A (1973–2015) into the SEER stage system. Localized, regional, and distant tumors were present in 91%, 6.4%, and 2.6% of patients, respectively. Basal diameter sizes ≤ and >14.9 mm were present in 2,950 (32.3%) and 796 (8.7%) patients, respectively. Tumor thickness >5.1 mm and ≤ 5.1 mm were present in 16.4% and 28.3% of patients, respectively. The rate of metastasis in the liver (1.2 %) was higher than that in the lungs (0.5%). Most patients had OM as their FMPI, and most were married (65.7%). In terms of treatment, 3,271 (35.9%), 6,181 (67.8%), and 247 (2.7%) patients had undergone surgery, radiotherapy, and chemotherapy, respectively. The median survival time was 132 months (Figure 2).

**Table 1:** Baseline demographics and clinical characteristics of patients (*n* = 9,120).

**Characteristic**		***N***
**Age**, ***y*** **(%)**		
	≤ 61	4,140 (45.4)
	>61	4,980 (54.6)
**Gender (%)**		
	Female	4,329 (47.5)
	Male	4,791 (52.5)
**Race (%)**		
	White	8,880 (97.4)
	Black	71 (0.8)
	Other	169 (1.9)
**Site (%)**		
	Choroid	7,139 (78.3)
	Ciliary body and iris	989 (10.8)
	Conjunctiva	574 (6.3)
	Other	418 (4.6)
**Laterality (%)**		
	Left	4,525 (49.6)
	Right	4,595 (50.4)
**Hist.type (%)**		
	Spindle	836 (9.2)
	Mixed	715 (7.8)
	Epithelioid	273 (3.0)
	NOS/other	7,296 (80.0)
**Stage (%)**		
	Localized	8,300 (91.0)
	Regional	583 (6.4)
	Distant	237 (2.6)
**Diameter (%)**		
	≤ 14.9 mm	2,950 (32.3)
	>14.9 mm	796 (8.7)
	Unknown	5,374 (58.9)
**Thickness (%)**		
	≤ 5.1 mm	2,582 (28.3)
	>5.1 mm	1,500 (16.4)
	Unknown	5,038 (55.2)
**Liver.met (%)**		
	No	5,267 (57.8)
	Yes	106 (1.2)
	Unknown	3,747 (41.1)
**Lung.met (%)**		
	No	5,328 (58.4)
	Yes	44 (0.5)
	Unknown	3,748 (41.1)
**FMPI (%)**		
	No	1,550 (17.0)
	Yes	7,570 (83.0)
**Marriage (%)**		
	Married	5,995 (65.7)
	Unmarried	1,369 (15.0)
	Divorced	789 (8.7)
	Widowed	967 (10.6)
**Surgery (%)**		
	No/unknown	5,849 (64.1)
	Yes	3,271 (35.9)
**Radiotherapy (%)**		
	No/unknown	2,939 (32.2)
	Yes	6,181 (67.8)
**Chemotherapy (%)**		
	No/unknown	8,873 (97.3)
	Yes	247 (2.7)

Hist.type, histologic subtype; Liver.met, metastasis at liver; Lung.met, metastasis at lung; FMPI, first malignant primary indicator.

### 3.2 Prognostic factor selection

The analysis included 16 independent variables: age, sex, race, site, laterality, histological subtype, stage, basal diameter, thickness, liver metastasis, lung metastasis, FMPI, marital status, surgery, radiotherapy, and chemotherapy. To select the independent prognostic factors for OM patients, we used the univariate and multivariate COX proportional hazards regression models (Table 2). In the univariate analysis, age >61 years (vs. age ≤ 61 years: HR = 2.680, 95% CI = 2.498–2.876, *P* < 0.001); sex male (vs. female: HR = 1.156, 95% CI = 1.084–1.233, *P* < 0.001); race, Blacks (vs. Caucasians: HR = 0.836, 95% CI = 0.668–1.387, *P* = 0.836), other (vs. Caucausians: HR = 1.179, 95% CI = 0.929–1.496, *P* = 0.176); site, ciliary body, and iris (vs. choroid: HR = 1.095, 95% CI = 0.989–1.212, *P* = 0.080), conjunctiva (vs. choroid: HR = 0.910, 95% CI = 0.791–1.046, *P* = 0.185), other (vs. choroid: HR = 1.872, 95% CI = 1.653–2.120, *P* < 0.001); laterality, right (vs. left: HR = 0.989, 95% CI = 0.927–1.055, *P* = 0.739); histological subtype, epithelioid (vs. spindle: HR = 2.801, 95% CI = 2.325–3.373, *P* < 0.001), mixed (vs. spindle: HR = 2.228, 95% CI = 1.924–2.579, *P* < 0.001), NOS/Other (vs. spindle: HR = 1.203, 95% CI = 1.07–1.352, *P* = 0.002); stage, regional (vs. localized: HR = 1.814, 95% CI = 1.615–2.039, *P* < 0.001), distant (vs. localized: HR = 7.321, 95% CI = 6.335–8.461, *P* < 0.001); basal diameter size, >14.9 mm (vs. ≤ 14.9 mm: HR = 2.618, 95% CI = 2.294–2.988, *P* < 0.001), unknown (vs. ≤ 14.9 mm: HR = 1.544, 95% CI = 1.412–1.687, *P* < 0.001); tumor thickness, > 5.1 mm (vs. ≤ 5.1 mm: HR = 2.016, 95% CI = 1.784–2.278, *P* < 0.001), unknown (vs. ≤ 5.1 mm: HR = 1.664, 95% CI = 1.511–1.833, *P* < 0.001); liver metastasis (vs. no liver metastasis: HR = 12.293, 95% CI = 9.905–15.255, *P* < 0.001), unknown (vs. no liver metastasis: HR = 1.161, 95% CI = 1.081–1.247, *P* < 0.001); lung metastasis (vs. no lung metastasis: HR = 8.795, 95% CI = 6.257–12.365, *P* < 0.001), unknown (vs. no lung metastasis: HR = 1.125, 95% CI = 1.048–1.208, *P* = 0.001); FMPI (vs. non-FMPI: HR = 0.574, 95% CI = 0.531–0.621, *P* < 0.001); marital status, unmarried (vs. married: HR = 0.996, 95% CI = 0.903–1.097, *P* = 0.929), divorced (vs. married: HR = 1.223, 95% CI = 1.090–1.372, *P* = 0.001), widowed (vs. married: HR = 2.121, 95% CI = 1.940–2.320, *P* < 0.001); surgery (vs. no/unknown surgery: HR = 1.641, 95% CI = 1.539–1.751, *P* < 0.001); radiotherapy (vs. no/unknown radiotherapy: HR = 0.592, 95% CI = 0.555–0.632, *P* < 0.001); chemotherapy (vs. no/unknown chemotherapy: HR = 1.818, 95% CI = 1.532–2.158, *P* < 0.001). We found that OM patients aged >61 years, predominantly men with lesions at other sites other than choroid, non-spindle types, non-localized lesions, basal diameter size >14.9 mm and unknown, tumor thickness >5.1 mm and unknown, metastasis to the liver and unknown, metastasis to the lung and unknown, marital status (divorced and widowed), and those who had undergone surgery/chemotherapy had a higher mortality risk. However, with OM as the FMPI, patients who were unmarried and had undergone radiotherapy showed improved OS. Our results indicated that race and laterality had no impact on OS in patients with OM.

**Table 2:** Univariate and multivariate analysis of overall survival for ocular melanoma patients.

**Characteristics**	**Univariate analysis (*****n*** **=** **9,120)**			**Multivariate analysis (*****n*** **=** **9,120)**		
	**HR**	**95%CI**	***P*** **-value**	**HR**	**95%CI**	***P*** **-value**
**Age**						
≤ 61	Reference			Reference		
>61	2.680	2.498–2.876	< 0.001^*^	2.463	2.286–2.654	< 0.001^*^
**Gender**						
Female	Reference			Reference		
Male	1.156	1.084–1.233	< 0.001^*^	1.249	1.167–1.337	< 0.001^*^
**Race**						
White	Reference					
Black	0.836	0.668–1.387	0.836			
Other	1.179	0.929–1.496	0.176			
**Site**						
Choroid	Reference			Reference		
Ciliary body and iris	1.095	0.989–1.212	0.080	0.992	0.894–1.101	0.879
Conjunctiva	0.910	0.791–1.046	0.185	0.523	0.444–0.614	< 0.001^*^
Other	1.872	1.653–2.120	< 0.001^*^	1.290	1.128–1.475	< 0.001^*^
**Laterality**						
Left	Reference					
Right	0.989	0.927–1.055	0.739			
**Hist.type**						
Spindle	Reference			Reference		
Epithelioid	2.801	2.325–3.373	< 0.001^*^	2.337	1.937–2.819	< 0.001^*^
Mixed	2.228	1.924–2.579	< 0.001^*^	1.685	1.453–1.955	< 0.001^*^
NOS/Other	1.203	1.070–1.352	0.002^*^	1.520	1.342–1.721	< 0.001^*^
**Stage**						
Localized	Reference			Reference		
Regional	1.814	1.615–2.039	< 0.001^*^	1.436	1.273–1.620	< 0.001^*^
Distant	7.321	6.335–8.461	< 0.001^*^	4.108	3.327–5.072	< 0.001^*^
**Diameter**						
≤ 14.9mm	Reference			Reference		
>14.9mm	2.618	2.294–2.988	< 0.001^*^	1.777	1.536–2.055	< 0.001^*^
Unknown	1.544	1.412–1.687	< 0.001^*^	1.180	1.012–1.377	0.035^*^
**Thickness**						
≤ 5.1mm	Reference			Reference		
>5.1mm	2.016	1.784–2.278	< 0.001^*^	1.512	1.320–1.732	< 0.001^*^
Unknown	1.664	1.511–1.833	< 0.001^*^	1.249	1.066–1.463	0.006^*^
**Liver.met**						
No	Reference			Reference		
Yes	12.293	9.905–15.255	< 0.001^*^	2.058	1.541–2.749	< 0.001^*^
Unknown	1.161	1.081–1.247	< 0.001^*^	0.975	0.138–6.905	0.980
**Lung.met**						
No	Reference			Reference		
Yes	8.795	6.257–12.365	< 0.001^*^	1.217	0.833–1.777	0.309
Unknown	1.125	1.048–1.208	0.001^*^	1.083	0.154–7.630	0.936
**FMPI**						
No	Reference			Reference		
Yes	0.574	0.531–0.621	< 0.001^*^	0.708	0.653–0.767	< 0.001^*^
**Marriage**						
Married	Reference			Reference		
Unmarried	0.996	0.903–1.097	0.929	1.158	1.049–1.279	0.004^*^
Divorced	1.223	1.090–1.372	0.001^*^	1.221	1.087–1.372	0.001^*^
Widowed	2.121	1.940–2.320	< 0.001^*^	1.539	1.397–1.695	< 0.001^*^
**Surgery**						
No/Unknown	Reference			Reference		
Yes	1.641	1.539–1.751	< 0.001^*^	1.227	1.113–1.354	< 0.001^*^
**Radiotherapy**						
No/Unknown	Reference			Reference		
Yes	0.592	0.555–0.632	< 0.001^*^	0.715	0.648–0.790	< 0.001^*^
**Chemotherapy**						
No/Unknown	Reference			Reference		
Yes	1.818	1.532–2.158	< 0.001^*^	1.588	1.323–1.905	< 0.001^*^

^*^P < 0.05.

Hist.type, histologic subtype; NOS, not otherwise specified; Liver.met, metastasis at liver; Lung.met, metastasis at lung; FMPI, first malignant primary indicator; HR, hazard ratio; CI, confidence interval.

We selected variables with *P* < 0.05 using the multivariate COX proportional hazards regression model. The final screening results of the multivariate analysis are as follows: age, age >61 years (vs. age ≤ 61 years: HR = 2.463, 95% CI = 2.286–2.654, *P* < 0.001); sex, male (vs. female: HR = 1.249, 95% CI = 1.167–1.337, *P* < 0.001); site, ciliary body and iris (vs. choroid: HR = 0.992, 95% CI = 0.894–1.101, *P* = 0.879), conjunctiva (vs. choroid: HR = 0.523, 95% CI = 0.444–0.614, *P* < 0.001), other (vs. choroid: HR = 1.290, 95% CI = 1.128–1.475, *P* < 0.001); histological subtype, epithelioid (vs. spindle: HR = 2.337, 95% CI = 1.937–2.819, *P* < 0.001), mixed (vs. spindle: HR = 1.685, 95% CI = 1.453–1.955, *P* < 0.001), NOS/other (vs. spindle: HR = 1.520, 95% CI = 1.342–1.721, *P* < 0.001); stage, regional (vs. localized: HR = 1.436, 95% CI = 1.273–1.620, *P* < 0.001), distant (vs. localized: HR = 4.108, 95% CI = 3.327–5.072, *P* < 0.001); basal diameter size, >14.9 mm (vs. ≤ 14.9 mm: HR = 1.777, 95% CI = 1.536–2.055, *P* < 0.001), unknown (vs. ≤ 14.9 mm: HR = 1.180, 95% CI = 1.012–1.377, *P* = 0.035); tumor thickness, > 5.1 mm (vs. ≤ 5.1 mm: HR = 1.512, 95% CI = 1.320–1.732, *P* < 0.001), unknown (vs. ≤ 5.1 mm: HR = 1.249, 95% CI = 1.066–1.463, *P* = 0.006); liver metastasis (vs. no liver metastasis: HR = 2.058, 95% CI = 1.541–2.749, *P* < 0.001), unknown (vs. no liver metastasis: HR = 0.975, 95% CI = 0.138–6.905, *P* = 0.980); lung metastasis (vs. no lung metastasis: HR = 1.217, 95% CI = 0.833–1.777, *P* = 0.309), unknown (vs. no lung metastasis: HR = 1.083, 95% CI = 0.154–7.630, *P* = 0.936); FMPI (vs. non-FMPI: HR = 0.708, 95% CI = 0.653–0.767, *P* < 0.001); marital status, unmarried (vs. married: HR = 1.158, 95% CI = 1.049–1.279, *P* = 0.004), divorced (vs. married: HR = 1.221, 95% CI = 1.087–1.372, *P* = 0.001), widowed (vs. married: HR = 1.539, 95% CI = 1.397–1.695, *P* < 0.001); surgery (vs. no/unknown surgery: HR = 1.227, 95% CI = 1.113–1.354, *P* < 0.001); radiotherapy (vs. no/unknown radiotherapy: HR = 0.715, 95% CI = 0.648–0.790, *P* < 0.001); chemotherapy (vs. no/unknown chemotherapy: HR = 1.588, 95% CI = 1.323–1.905, *P* < 0.001). Briefly, age, sex, site, histological subtype, stage, basal diameter size, tumor thickness, metastasis to the liver, FMPI, marital status, and surgical intervention/radiotherapy/chemotherapy were independent prognostic factors for OS (all *P* < 0.05).

### 3.3 Nomogram model for OS prognosis

**Figure 3:**
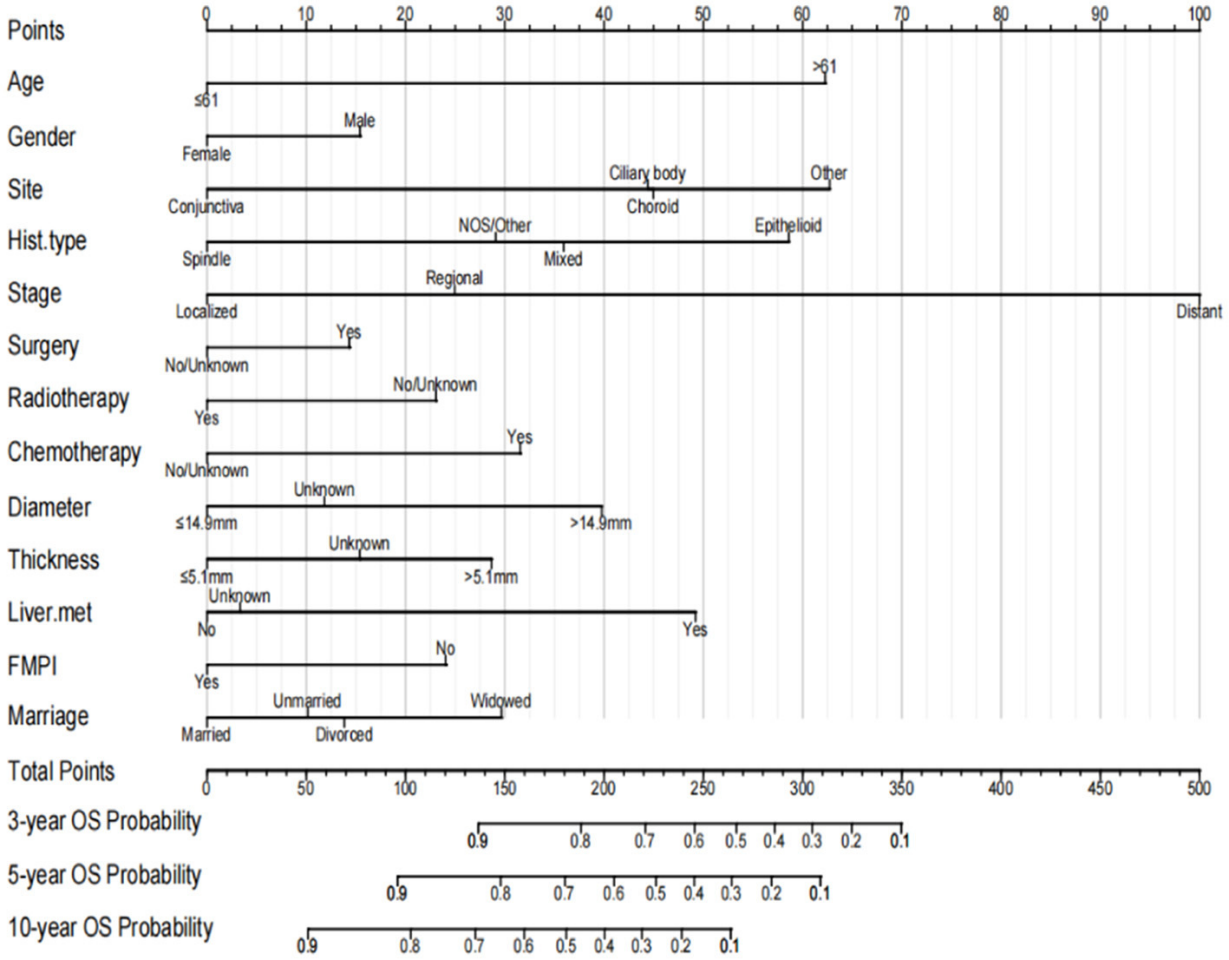
Prognostic nomogram for predicting the probability of 3-, 5- and 10-year OS in patients with ocular melanoma.

A nomogram was constructed by incorporating all independent factors to forecast the survival probability of patients with OM at 3, 5, and 10 years (Figure 3). As shown in the figure, the stage of OM had the most significant influence on survival, followed by the tumor site, age, histological subtype, liver metastasis, tumor diameter, chemotherapy, marital status, tumor thickness, FMPI, radiotherapy, sex, and surgery. Each factor in the nomogram was assigned a specific score using a point system. The scores for all factors were summed and a vertical line was drawn to determine the total score, indicating the survival probability of patients with OM at 3, 5, and 10 years.

### 3.4 Model performance

**Figure 4:**
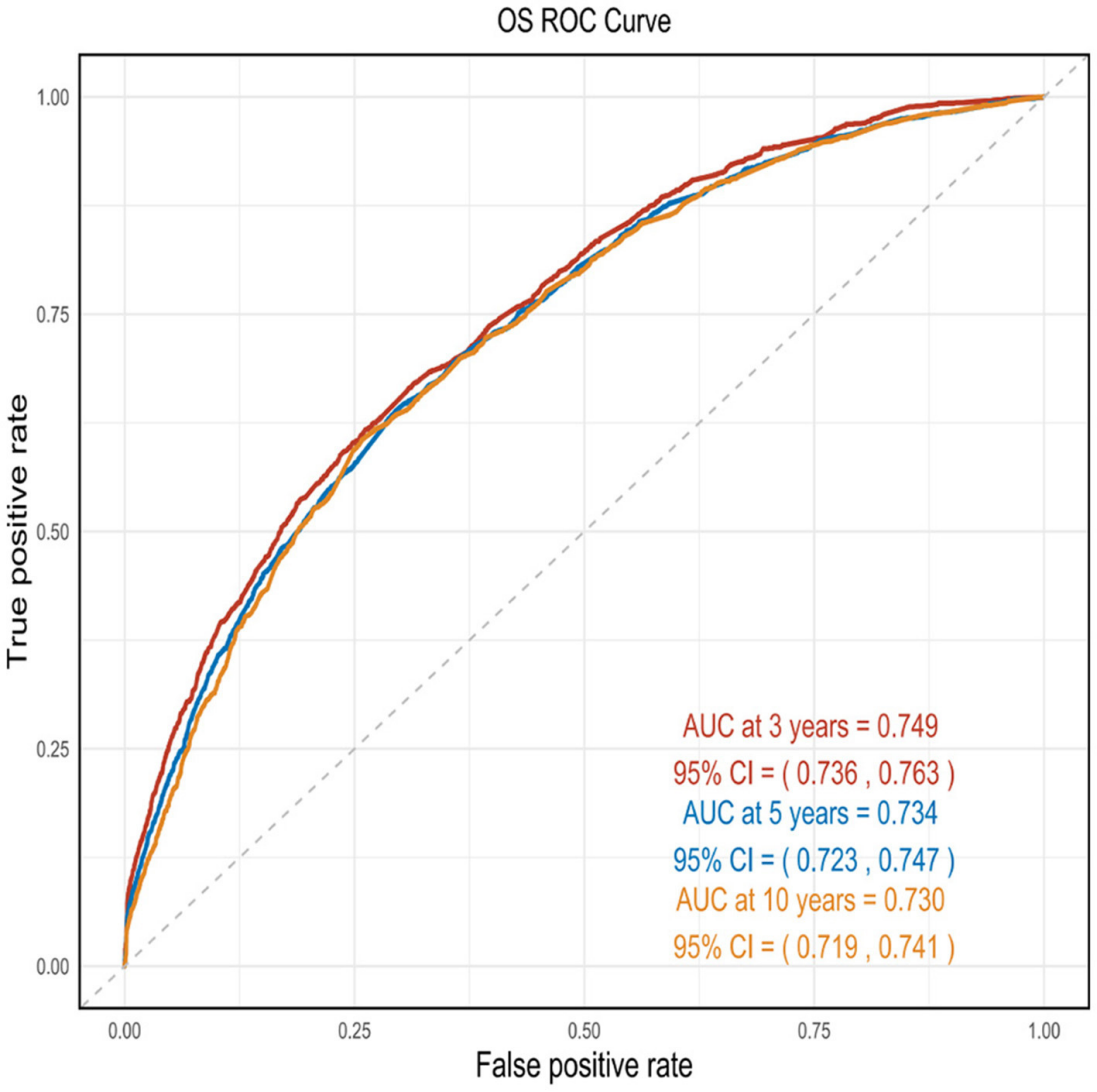
ROC curves were used to verify the predictability of the nomogram at different thresholds, and the AUCs.

**Figure 5:**
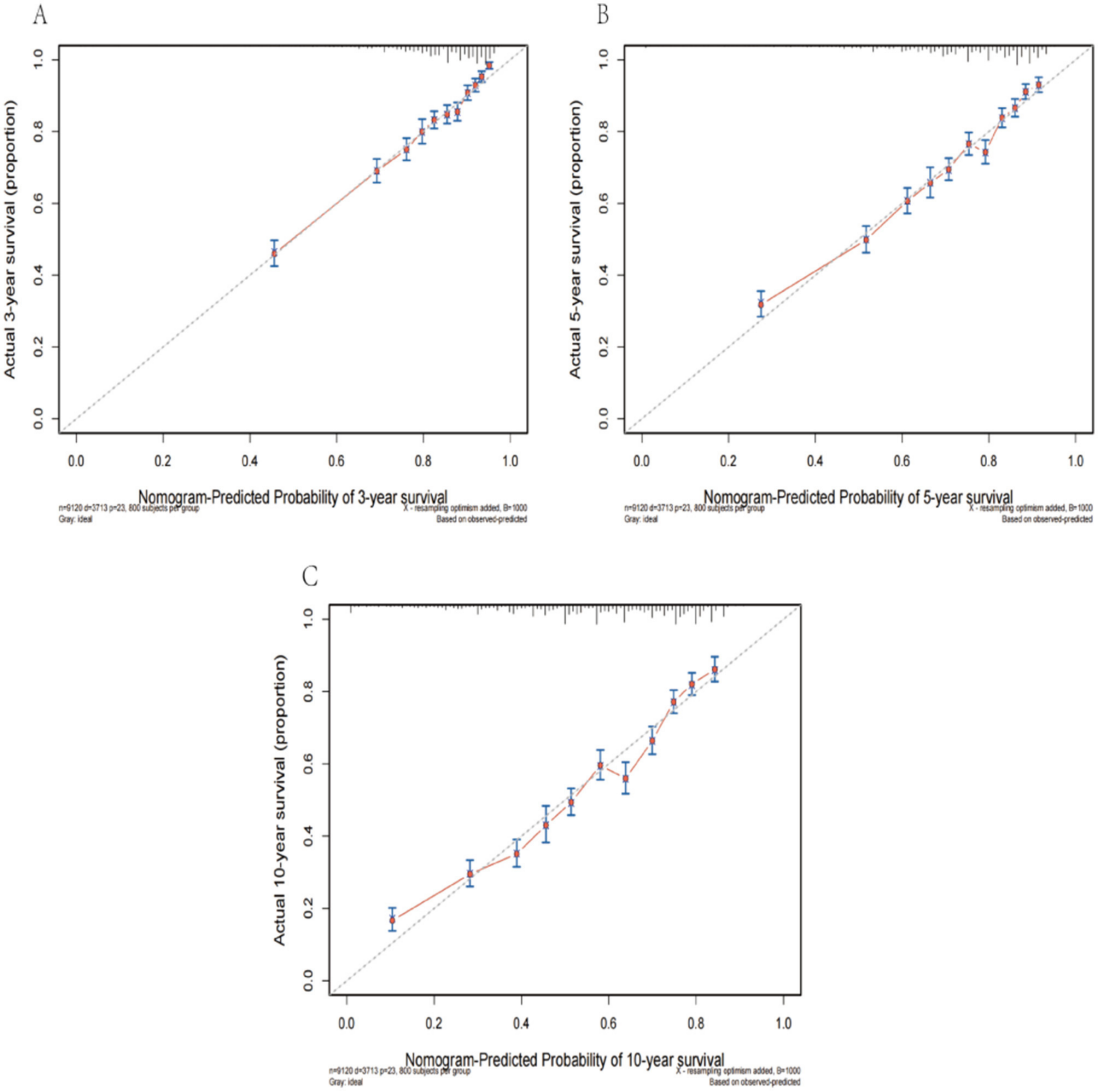
The calibration plots predicting 3- **(A)**, 5- **(B)**, and 10-year **(C)** survival in patients with OM were close to the ideal curves.

**Figure 6:**
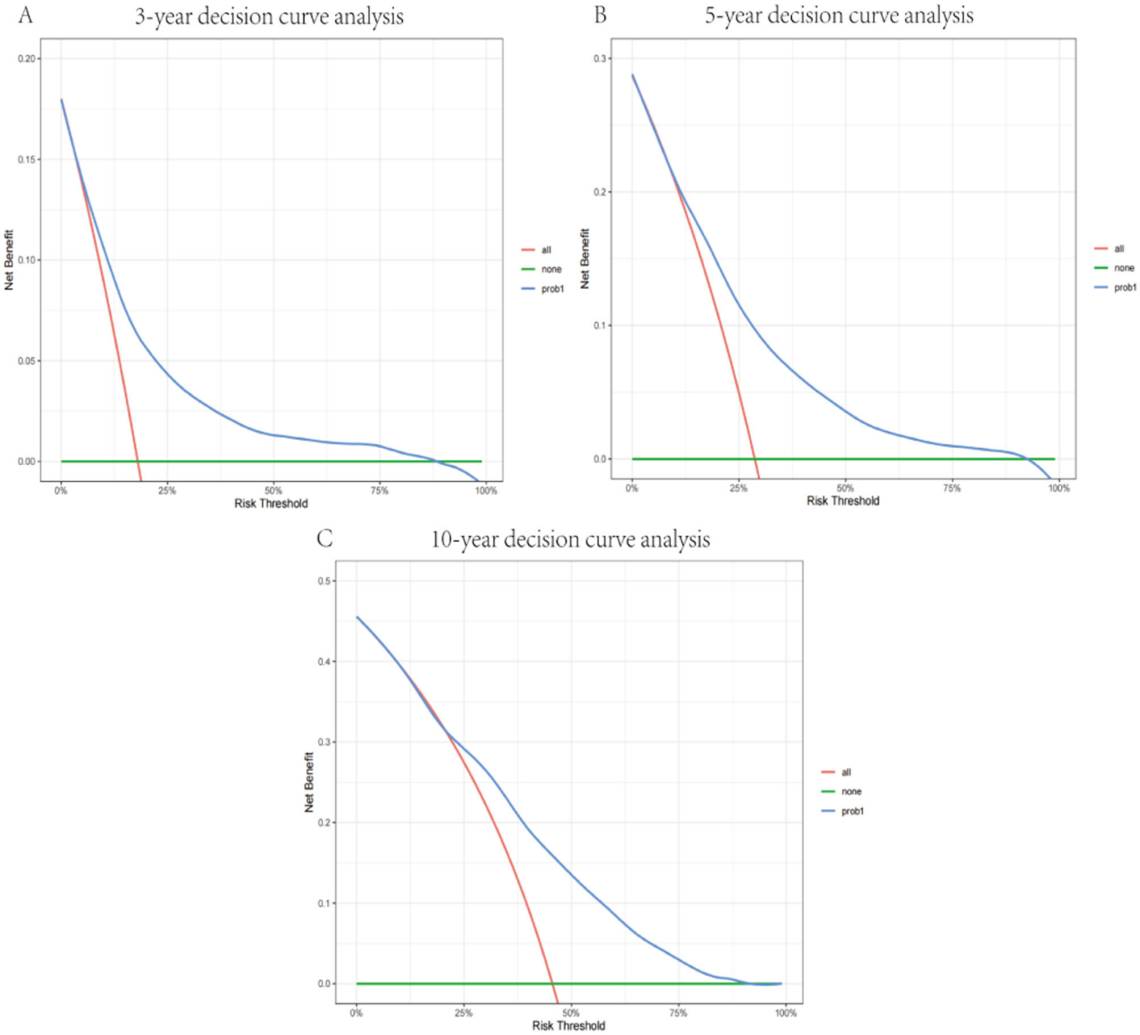
Decision curve of the model in predicting 3- **(A)**, 5- **(B)**, and 10-year **(C)** prognosis. The *blue curve* represents the net benefits of the model for predicting outcomes.

This nomogram had an optimal C-index of 0.712 (95% CI = 0.702–0.722), indicating satisfactory discrimination ability of the survival analysis model. We evaluated the performance of the nomogram in predicting 3-, 5-, and 10-year survival probabilities in patients with OM. ROC curves were used to verify the predictability of the nomogram at different thresholds, and the AUCs were computed for the 3-, 5-, and 10-year survival rates (0.749, 0.734, and 0.730, respectively), and the 95% CI were (0.736–0.763), (0.723–0.747), and (0.719–0.741), respectively (Figure 4). The calibration plots predicting 3-, 5-, and 10-year OS in patients with OM were close to the ideal curves, showing good agreement between the predicted probabilities and the actual observed results (Figure 5). DCA is a novel evaluation tool used to evaluate the net benefit of a predictive model under different thresholds and determine the usefulness of the model in clinical decision-making. The curve demonstrated a superior net benefit, highlighting its significant clinical utility in guiding prognostic assessments (Figure 6). We verified the predictive ability of the proposed model from multiple dimensions.

### 3.5 Validation of the nomogram

**Figure 7:**
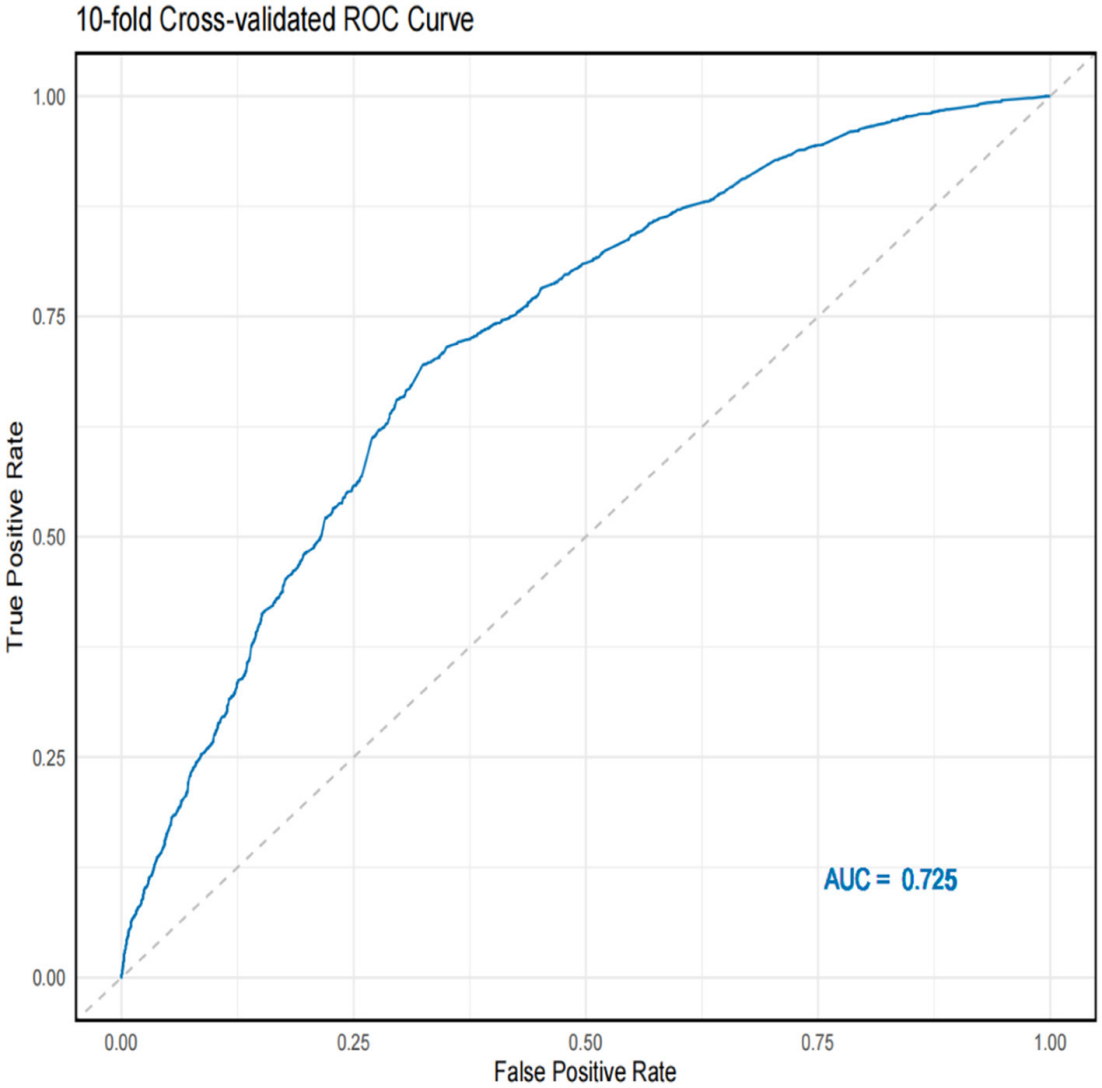
10-fold cross-validation ROC curve and AUC value.

Using 10-fold cross-validation, we assessed the predictive performance of the nomogram. The ROC curve indicated that the model had good discriminatory power for predicting survival status (Figure 7). The average AUC value obtained from cross-validation was 0.725 (95% CI = 0.711–0.732).

### 3.6 Machine learning

**Figure 8:**
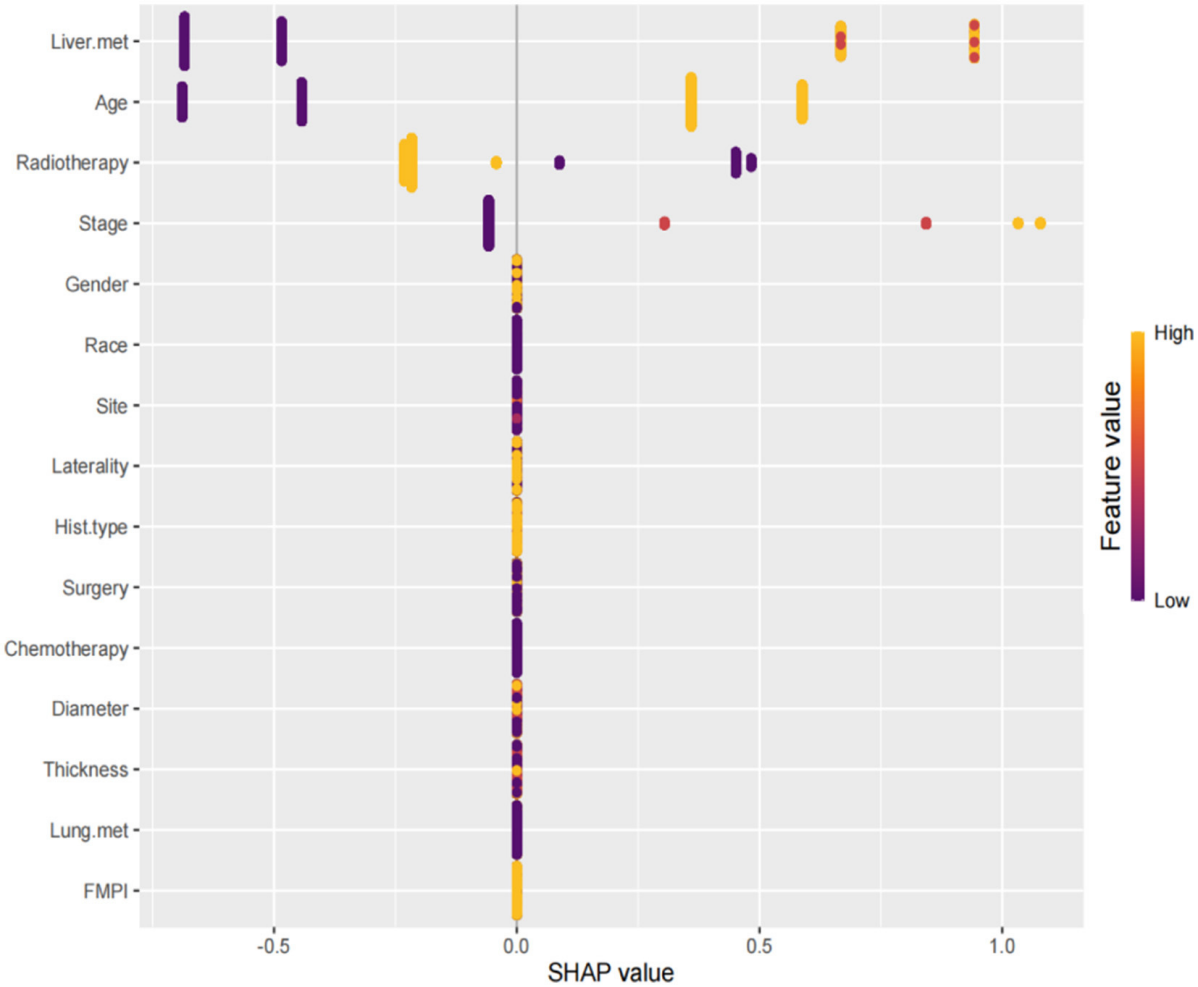
SHAP summary plot. The plot ranked the factors by their importance, with liver metastasis being the most influential, followed by age, radiotherapy, stage, and other factors.

**Figure 9:**
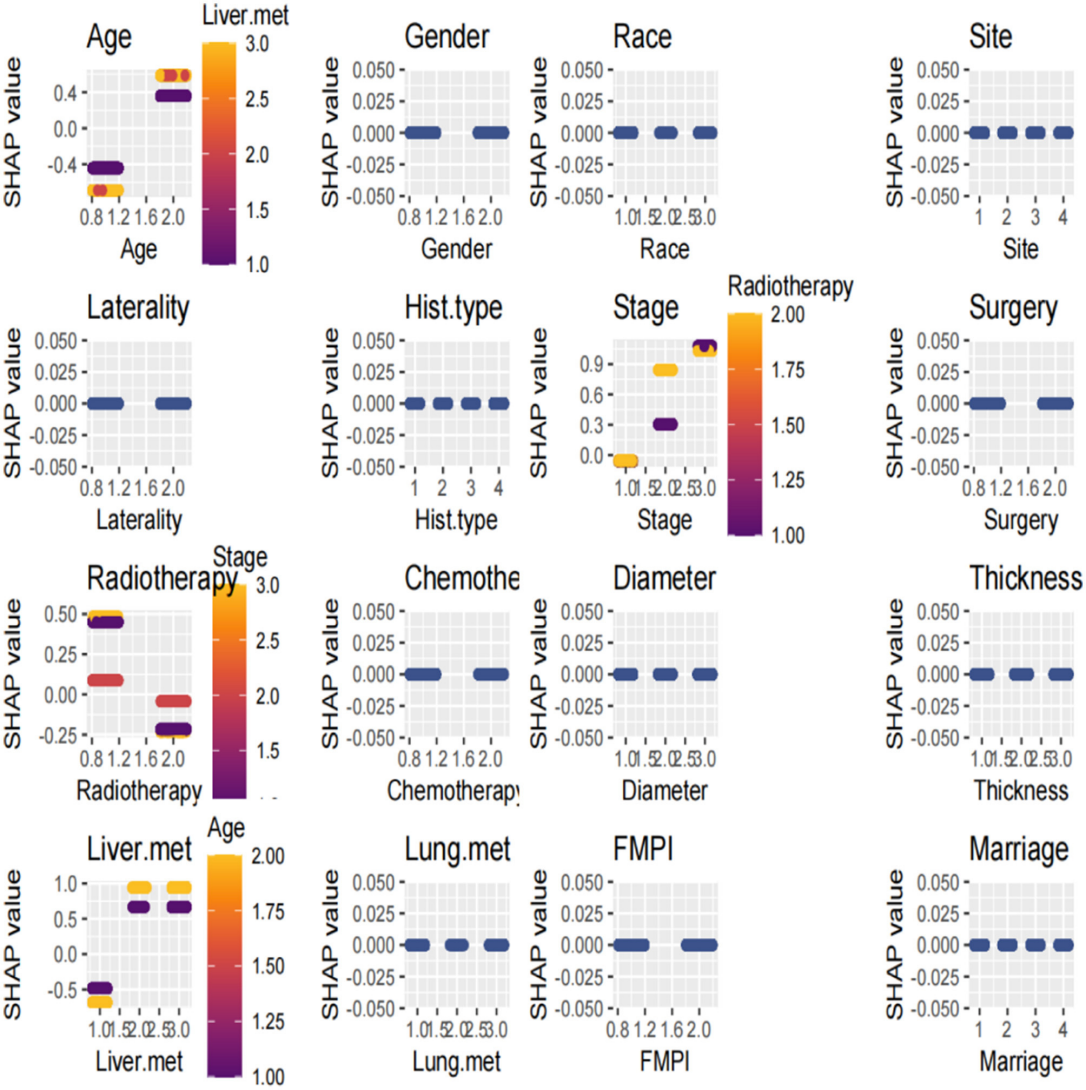
The multivariate dependence plot demonstrated the interactions between the different variables.

**Figure 10:**
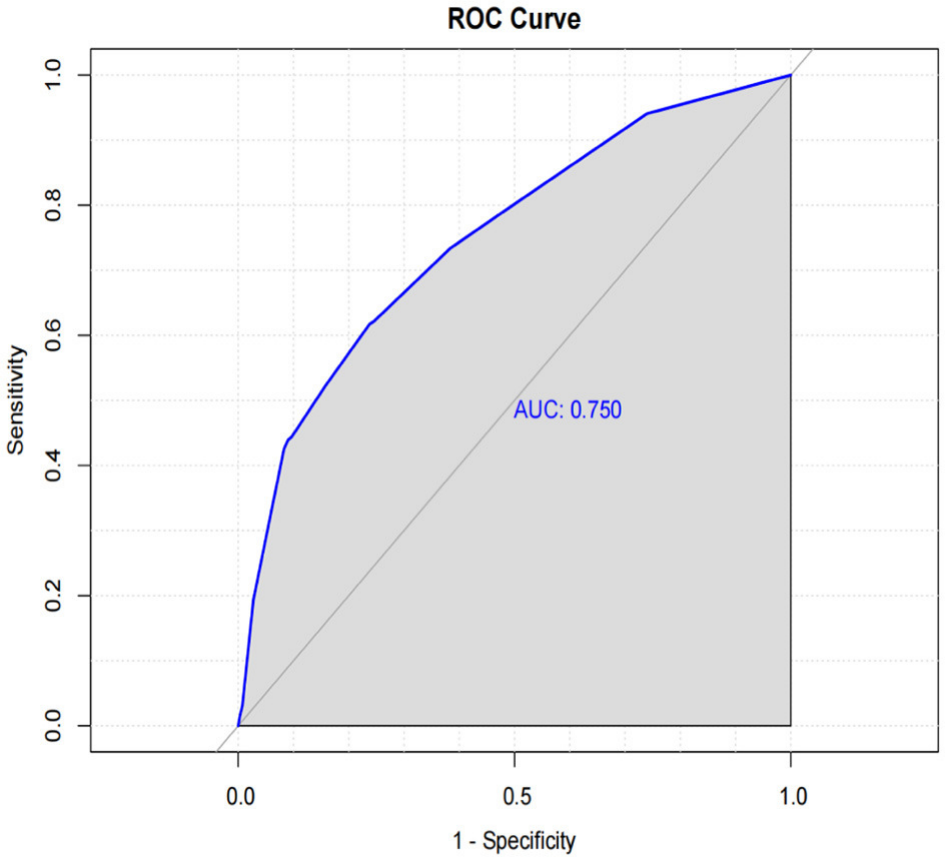
The ROC curve to verify the predictability of the machine learning model.

The SHAP summary plot is a visualized tool to rank the characteristics in the model and the influence of each feature on the predictive ability of the model. The plot ranked the factors by their importance, with liver metastasis being the most influential, followed by age, radiotherapy, stage, and other factors (Figure 8). These important factors were then incorporated into the nomogram. The multivariate dependence plot demonstrated the interactions between the different variables (Figure 9). Older patients with liver metastases and more advanced tumor stages who had undergone radiotherapy had a significant impact on the model predictions. Interactions between other variables had no significant impact on the model predictions. The ROC curves used to verify the predictability of the machine learning model showed an AUC of 0.750 (95% CI = 0.729–0.766), indicating good predictability (Figure 10).

## 4 Discussion

In this study, we established a prognostic model for predicting the survival probability of patients with OM. The model incorporated 13 significant independent prognostic variables, including age, sex, tumor site, histologic subtype, stage, basal diameter, tumor thickness, liver metastasis, FMPI, marital status, and treatment with surgery/radiotherapy/chemotherapy. Previous studies have not consistently analyzed age and OS (5, 12). We used the X-tile software to determine the best cutoff value for age and found that patients aged ≥61 years had a worse prognosis. Our findings revealed that men had a shorter OS than women, corroborating the findings of previous studies (5, 13, 14). In a retrospective study of 8,033 patients, increased melanoma thickness was associated with an increased risk of metastasis. Each additional millimeter of thickness increased the risk of metastasis by 5% after 10 years (15). Furthermore, we included tumor thickness as a variable in our nomogram. Consistent with those of previous studies, our findings indicated that other factors related to the primary tumor, including tumor location, diameter, histological subtype, and distant metastasis, were independent prognostic factors (8, 16–18). In addition, our study identified FMPI and marital status as factors influencing survival. In our study on the treatment modalities affecting the survival prognosis of patients with OM, the risk of mortality was lower among patients who had undergone radiotherapy than among those who had undergone surgical intervention or chemotherapy. Compared with previous studies, this study identified more prognostic factors for predicting the survival of patients with OM. Because UM accounts for ~85% of all malignant OMs, most studies have established prediction models for UM (19). Unlike previous studies that targeted UM specifically, we established a survival prediction model for all patients with malignant OM, regardless of the specific site of the tumor. Given the rarity of OM, external validation of large amounts of data using this nomogram was challenging. Nevertheless, we conducted calibration and internal validation using multiple dimensions to evaluate the model prediction. The nomogram exhibited strong predictive performance for survival in patients with OM, with a C-index and AUC > 0.7. The use of the ROC curve, calibration curve, and DCA further validates its accuracy. The 10-fold cross-validation method provides a robust and reliable estimate of the model's performance (20). By repeatedly partitioning the dataset and evaluating the model on different subsets, we ensured consistent performance across different subsets of the data and were not overly dependent on a specific subset of the data (21). The AUC value of the 10-fold cross-ROC curve was also > 0.7, indicating good performance. This method is particularly effective in preventing overfitting and provides confidence that the model will generalize well to new, unseen data (22). In addition to the calibration and verification methods mentioned above, we introduced an innovative machine-learning algorithm for comparison with a nomogram.

In recent years, machine learning has emerged as a crucial tool for cancer prediction because it can learn from clinical data and construct accurate prediction models (23). However, the challenge with machine learning models lies in their black-box nature, which limits their application in clinical practice (24). XGBoost is an efficient gradient-boosting algorithm that has shown promising results in large-scale datasets (25, 26). SHAP, an interpretable machine-learning method, has been widely used to elucidate the prediction outcomes of complex models (25). SHAP assigns an importance weight to each feature, revealing its contribution to the model's prediction. The SHAP method combined with the XGBoost model revealed that liver metastasis, age, radiotherapy, and tumor stage had the most significant effects on the model. These variables were also included in the nomogram, demonstrating strong consistency between the machine prediction model and the nomogram. The SHAP value effectively captured the complex interaction effects and non-linear relationships (27). The ROC of our machine learning model showed good performance, with an AUC of 0.750. Additionally, the multivariate dependency graph highlighted that the interactions between age and liver metastasis, stage, and radiotherapy had the greatest impact on survival prediction.

We observed disparities in the most influential variables in both the nomogram and the machine learning models. The nomogram model identified the stage as the foremost determinant of survival prognosis, whereas the machine learning model prioritized liver metastasis as a pivotal variable. SHAP is rooted in the principles of game theory and provides a precise quantification of each feature's influence on the model output (27). Consequently, the significance of staging in the nomogram model may encompass the contribution of liver metastasis variables. Therefore, integrating conventional techniques with innovative machine learning approaches is crucial. Further exploration through deep learning analysis is warranted to achieve an optimal amalgamation of these methodologies for clinical applications.

Our study had certain limitations. The prediction model was developed based on the retrospective data, which inevitably introduced bias. Owing to database limitations, other variables that may impact survival prognosis, including Eastern Cooperative Oncology Group performance and new treatments such as targeted therapy and immunotherapy, were not collected. These variables should also be analyzed. The survival events in this study considered only OS, necessitating the inclusion of cancer-specific survival in future studies for comparative analysis. During the variable selection process, we acknowledged that brain and bone metastases may influence survival. However, the sample sizes for these variables were small, resulting in an unstable model. Incorporation of more patient data in further analyses is necessary to enhance the robustness of the model. Given that OM is a rare disease, accumulating a larger sample size and clinical information is crucial for reliable model training and external validation. Molecular pathological features, particularly genetic characteristics, can significantly affect the survival of patients with OM. Therefore, we aimed to combine clinicopathological features with genomic variables to construct a more accurate prognostic prediction model. Additionally, with advancements in artificial intelligence, these tools can be leveraged alongside traditional methods to develop an optimal model for predicting survival in rare diseases, such as OM.

## 5 Conclusion

In conclusion, we developed a nomogram incorporating 13 significant clinicopathological variables to predict survival in patients with OM. The synthesis of ROC and AUC values, calibration plots, DCA, 10-fold cross-validation, and machine learning models with SHAP values demonstrated the robust performance of the nomogram. These findings enhance our understanding of the prognosis of OM and assist clinicians in making informed decisions.

## Data Availability

The datasets presented in this study can be found in online repositories. The names of the repository/repositories and accession number(s) can be found below: Data regarding the SEER program can be found on the SEER website (https://seer.cancer.gov).
